# Pediatric cancer predisposition syndromes involving non-central nervous system solid pediatric tumors: a review on their manifestations with a focus on histopathology

**DOI:** 10.1007/s00428-025-04029-1

**Published:** 2025-01-23

**Authors:** B. Schurink, M. Reyes-Múgica, R. R. de Krijger

**Affiliations:** 1 Department of Pathology, Amsterdam University Medical Centers, Location VUmc. De Boelelaan 1117, 1081 HV Amsterdam, The Netherlands; 2 Princess Máxima Center for Pediatric Oncology, Heidelberglaan 25, 3584 CS Utrecht, The Netherlands; 3 Department of Pathology & Laboratory Medicine, University of Miami Miller School of Medicine, 5301 South Congress Avenue Atlantis, Miami, FL 33462 USA; 4 Department of Pathology, University Medical Center Utrecht, Heidelberglaan 100, 3584 CX Utrecht, The Netherlands

**Keywords:** Pediatrics, Cancer predisposition syndrome, Histology, Pathology

## Abstract

Germline genetic alterations and their associated cancer predisposition syndromes (CPS) are an important cause of pediatric cancer. Early recognition is of great importance for targeted surveillance, early detection, and prompt (personalized) therapeutic interventions. This review provides an overview of non-central nervous system solid pediatric tumor types, in relation to their associated CPS, with an emphasis on their histology. It serves as a guide for (pediatric) pathologists to increase their awareness of histological clues that suggest a CPS and warrant referral to the clinical geneticist.

## Introduction

Although relatively rare, pediatric cancers place a significant burden on affected children and their families. Germline genetic alterations and their associated cancer predisposition syndromes (CPS) are an important cause of cancer in young patients, with a reported contribution of 10% [1]. CPS encompass a diverse spectrum of conditions, each with unique clinical manifestations and cancer susceptibilities. Prompt recognition of these syndromes is of great importance; it has not only revolutionized our understanding of tumorigenesis, but also provides invaluable opportunities for targeted surveillance, early detection, and personalized therapeutic interventions.

Adequate histologic diagnosis is the cornerstone to characterize and classify pediatric malignancies, and a crucial element of decision-support tools that aid the clinician in identifying patients at high risk of an underlying CPS (e.g., MIPOGG [2]). Histology also facilitates a tailored therapeutic approach based on tumor type, grade, and molecular characteristics. Furthermore, it may provide the first clue to identify a CPS. Therefore, it is of great importance for the pathologist to recognize the different pediatric tumor types and to be aware of their underlying genetic aberrations.

**Fig. 1:**
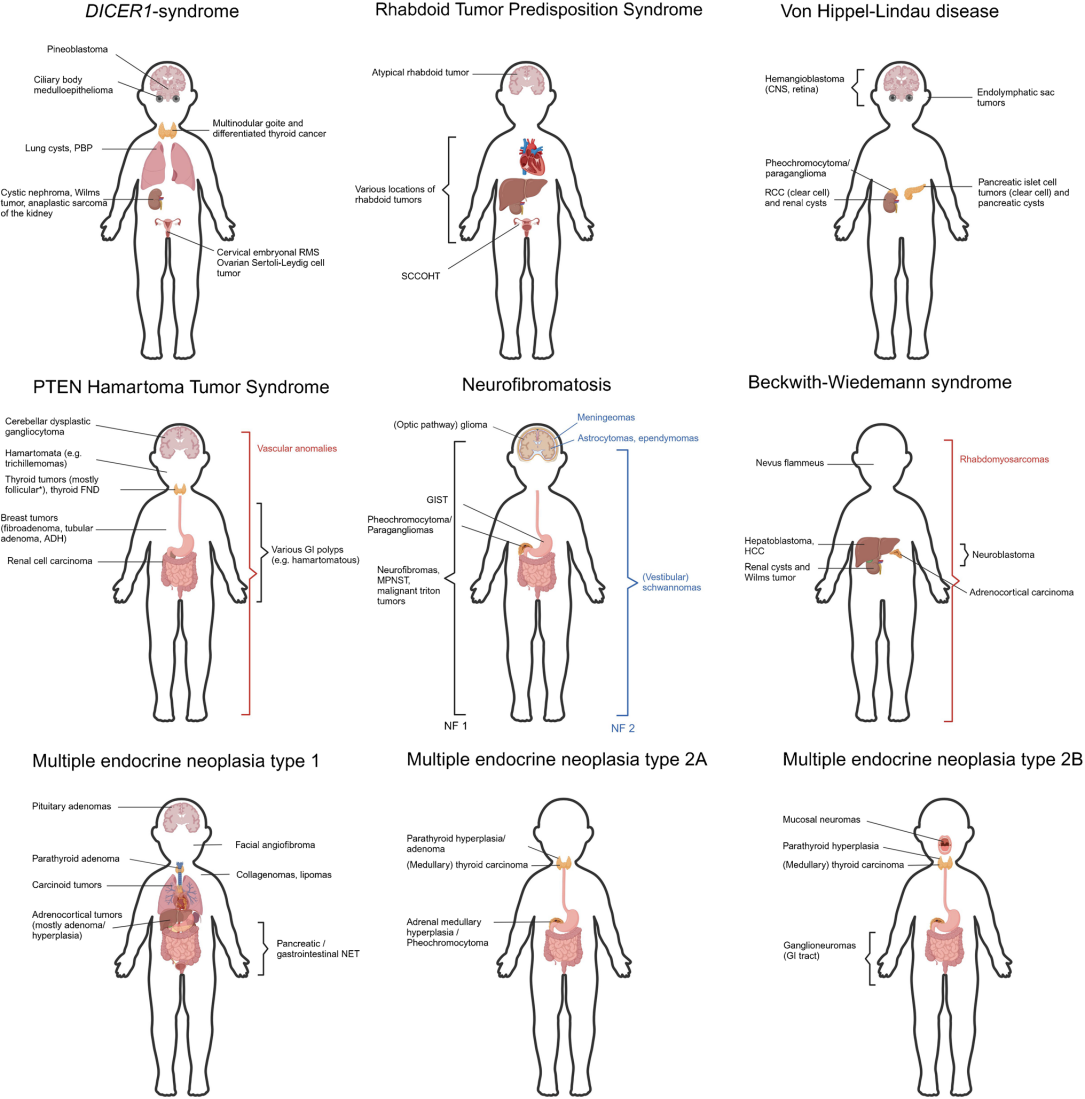
Schematic representation of solid tumor types in selected cancer predisposition syndromes (CPS) with multiple solid tumor types occurring in the pediatric age range. Abbreviations. ADH, atypical ductal hyperplasia; FND, follicular nodular disease; HCC, hepatocellular carcinoma; GI, gastrointestinal; GIST, gastrointestinal stromal tumor; MPNST, malignant peripheral nerve sheath tumor; NET, neuroendocrine tumor; NF, neurofibromatosis

This review discusses the most important non-central nervous system solid pediatric tumors with their associated predisposition syndrome(s) and provides essential histologic clues to the diagnosis. While some tumors directly point towards a CPS (such as pleuropulmonary blastoma and *DICER1*-syndrome), others are part of multiple CPS or occur sporadically, and, certain histological clues might suggest a hereditary condition (e.g., C-cell hyperplasia in the thyroid gland in the context of multiple endocrine neoplasia type 2). The tumor types will be arranged according to their corresponding organ system, to mirror the daily pathology practice as closely as possible. Figure 1 shows the most important CPS and their associated tumors. A list of the most important CPS and their associated genes is presented in Table 1.

**Table 1:** Pediatric cancer predisposition syndromes and their type of inheritance and associated genes/chromosomal alterations

	Cancer predisposition syndrome	Type of inheritance	Genes/chromosomal alterations
Peripheral neuroblastic tumors			
Neuroblastoma [4]	ALK-associated syndrome	Autosomal dominant	*ALK*
	Beckwith-Wiedemann	Imprinting and growth abnormalities	Various genetic or epigenetic defects on chromosome 11p15.5
	Congenital central hypoventilation	Autosomal dominant	*PHOX2B*
	Costello syndrome	Autosomal dominant	*HRAS*
	Fanconi anemia	Autosomal recessive	*BRIP1, BRCA2, PALB2, FANCA, FANCC, FANCG *etc
	Li-Fraumeni syndrome	Autosomal dominant	*TP53 [especially R337H]*
	Neurofibromatosis type 1	Autosomal dominant	*NF1*
	Noonan syndrome	Autosomal dominant, except LZTR1 which is recessive	*PTPN11, SOS1, RAF1, RIT1, KRAS, NRAS, BRAF, MAP2KI, LZTR1*
	ROHADD syndrome	Unknown	Unknown
	Weaver syndrome	Autosomal dominant	*EZH2*
Soft tissue tumors			
Osteosarcoma [6]	ATR-X syndrome	X-linked	
	Bloom syndrome	Autosomal recessive	*RECQL3 (BLM)*
	Diamond-Blackfan anemia	Autosomal dominant	*RP genes*
	Hereditary retinoblastoma	Autosomal dominant	*Rb1*
	Li-Fraumeni	Autosomal dominant	*TP53*
	Rothmund-Thomson syndrome	Autosomal recessive	*RECQL4*
	Werner syndrome	Autosomal recessive	*WRN*
Ewing sarcoma [6]	Hereditary retinoblastoma	Autosomal dominant	*Rb*
	FANCC	Autosomal recessive	*FANCC*
	BRCA	Autosomal dominant	*BRCA1, BRCA2*
Rhabdomyosarcomas/DICER1-related sarcomas [7]	DICER1 syndrome	Autosomal dominant	*DICER1*
	Li-Fraumeni	Autosomal dominant	*TP53*
	Beckwith-Wiedemann	Imprinting and growth abnormalities	Various genetic or epigenetic defects on chromosome 11p15.5
	Costello syndrome	Autosomal dominant	*HRAS*
	Mosaic variegated aneuploidy	Autosomal recessive (in most cases)	*BUB1B, CEP57*
	Neurofibromatosis type 1	Autosomal dominant	*NF1*
	Nijmegen breakage syndrome	Chromosomal instability	*NBN*
	Noonan syndrome	Autosomal dominant, except LZTR1 which is recessive	*PTPN11, SOS1, RAF1, RIT1, KRAS, NRAS, BRAF, MAP2KI, LZTR1*
Leiomyomas [5]	Hereditary leiomyomatosis and renal cell cancer	Autosomal dominant	*FH*
Desmoid-type fibromatosis [14]	Familial adenomatous polyposis syndrome (FAP), including Gardner syndrome	Autosomal dominant	*APC*
Gastrointestinal stromal tumor (GIST) [18]	Neurofibromatosis type 1	Autosomal dominant	*NF1*
	SDH deficiency syndrome	Autosomal dominant (SDHD with paternal inheritance)	*MAX, SDHx* genes,* TMEM127, FH*
	Familial GIST syndromes	Autosomal dominant	*KIT, PDGFRA*
Schwannoma [19]	Carney complex	Autosomal dominant	*CNC1 and 2, PRKAR1A*
	Neurofibromatosis type 2	Autosomal dominant	*NF2*
	Schwannomatosis	Sporadic, in 15–25% of cases autosomal dominant	*SMARCB1, NF2*
Neurofibroma [19]	Neurofibromatosis (mostly type 1)	Autosomal dominant	*NF1*
Malignant nerve sheath tumor [19]	Neurofibromatosis type 1	Autosomal dominant	*NF1*
	Li-Fraumeni syndrome	Autosomal dominant	*TP53*
Genital system			
Gonadoblastoma [22, 23]	Denys-Drash	Autosomal dominant	*WT1*
	Frasier syndrome	Autosomal dominant	*WT1* (intron 9)
	Swyer syndrome	Autosomal dominant or autosomal recessive	*SRY* (yp11.3), *DHH* (12q13.1), *NR5A1* (9q33), *CBX2* (17q25), *DMRT* 1,2 (9p24.3)
	Simpson-Golabi-Behmel syndrome	X-linked	*GPC3*
	Turner syndrome	Structural chromosomal	Sex chromosomes
	WAGR syndrome	Structural chromosomal	11p13 deletion, WT1
Extracranial germ cell tumors [26–28]	Klinefelter syndrome	Structural chromosomal	Sex chromosomes
	Schinzel-Giedion syndrome	Autosomal dominant	*SETBP1* (gain of function)
	Swyer syndrome	Autosomal dominant or autosomal recessive	*SRY* (yp11.3), *DHH* (12q13.1), *NR5A1* (9q33), *CBX2* (17q25), *DMRT* 1,2 (9p24.3)
	Currarino syndrome	Autosomal dominant	*MNX1*
Small-cell carcinoma of the ovary hypercalcemic type [32]	Rhabdoid predisposition syndrome type 2	Autosomal dominant	*SMARCA4*
Ovarian Sertoli-Leydig cell tumors [34]	DICER1 syndrome	Autosomal dominant	*DICER1*
Ovarian fibroma [37]	Nevoid basal cell carcinoma syndrome (Gorlin-Goltz syndrome)	Autosomal dominant	*PTCH1, SUFU*
Urinary system			
Nephroblastoma [38]	Beckwith-Wiedemann syndrome	Imprinting and growth abnormalities	Various genetic or epigenetic defects on chromosome 11p15.5
	Bloom syndrome	Autosomal recessive	*RECQL3 (BLM)*
	Bohring-Opitz syndrome	Autosomal dominant	*ASXL1*
	Constitutional mismatch repair deficiency syndrome	Autosomal recessive	MLH1, MSH2, MSH6, PMS2
	DICER1-syndrome	Autosomal dominant	*DICER1*
	Familial Wilms tumor	Autosomal dominant (in case of TRIM28 with maternal inheritance)	*CTR9, HACE1, REST, TRIM28*
	Fanconi anemia	Autosomal recessive	*BRIP1, BRCA2, PALB2, FANCA, FANCC, FANCG *etc
	Hyperparathyroidism-jaw tumor syndrome	Autosomal dominant	*HRPT2*
	Mosaic variegated aneuploidy	Autosomal recessive (in most cases)	
	Perlman syndrome	Autosomal recessive	*DIS3L2*
	Simpson-Golabi-Behmel syndrome	X-linked	*GPC3 and 4*
	Sotos syndrome	Autosomal dominant	*NSD1*
	WT1-associated syndromes (e.g., Denys-Drash syndrome, Frasier syndrome, WAGR syndrome)	See the respective syndromes elsewhere in the table	See the respective syndromes elsewhere in the table
Renal cell carcinoma [43]	Von Hipple Lindau	Autosomal dominant	*VHL*
	Hereditary papillary renal cell carcinoma	Autosomal dominant	*MET*
	Hereditary leiomyomatosis and renal cell cancer	Autosomal dominant	*FH*
	PTEN-hamartoma tumor syndrome	Autosomal dominant	*PTEN*
	SDH deficiency syndrome	Autosomal dominant (SDHD with paternal inheritance)	*MAX, SDHx* genes,* TMEM127, FH*
	Birt-Hogg-Dubé	Autosomal dominant	*FLCN*
	Tuberous sclerosis complex	Autosomal dominant	*TSC1 and 2*
Malignant rhabdoid tumor of the kidney [45]	Rhabdoid Tumor Predisposition syndrome (type 1)	Autosomal dominant	*SMARCB1*
Digestive tract tumors			
Hepatoblastoma [47, 48]	Beckwith-Wiedemann syndrome	Imprinting and growth abnormalities	Various genetic or epigenetic defects on chromosome 11p15.5
	Familial adenomatous polyposis syndrome (FAP), including Gardner syndrome	Autosomal dominant	*APC*
Hepatocellular carcinoma [50–52]	Alagille syndrome	Autosomal dominant	*JAG1* (20p12) (AGS type 1), *NOTCH 2* (AGS type 2)
	Carney complex	Autosomal dominant	*CNC1 and 2, PRKAR1A* inactivation
	Caroli disease	Not certain (autosomal recessive/autosomal dominant)	*PKHD1*
	Simpson-Golabi-Behmel syndrome	X-linked	*GPC3 and 4*
Pancreatoblastoma [55]	Beckwith-Wiedemann syndrome	Imprinting and growth abnormalities	Various genetic or epigenetic defects on chromosome 11p15.5
	Von Hipple Lindau	Autosomal dominant	*VHL*
Neuroendocrine neoplasms [57]	Multiple endocrine neoplasia 1	Autosomal dominant	*MEN1*
	Multiple endocrine neoplasia 2	Autosomal dominant	*RET*
	Neurofibromatosis type 1	Autosomal dominant	*NF1*
	Von Hipple Lindau	Autosomal dominant	*VHL*
Endocrine system			
Thyroid tumors, follicular cell derived tumors [59]	Carney complex	Autosomal dominant	*CNC1 and 2, PRKAR1A* inactivation
	*DICER1* syndrome	Autosomal dominant	*DICER1*
	Familial adenomatous polyposis syndrome (FAP), including Gardner syndrome	Autosomal dominant	*APC*
	Li-Fraumeni syndrome	Autosomal dominant	*TP53*
	PTEN-hamartoma tumor syndrome	Autosomal dominant	*PTEN*
Medullary thyroid carcinomas [59]	Multiple endocrine neoplasia type 2	Autosomal dominant	*RET*
Multinodular goiter [63]	DICER1	Autosomal dominant	DICER1
Parathyroid neoplasms [66]	Hyperparathyroidism-jaw tumor syndrome	Autosomal dominant	*HRPT2, CDC73*
	Multiple endocrine neoplasia type 1	Autosomal dominant	*MEN1*
	Multiple endocrine neoplasia type 2	Autosomal dominant	*RET*
	Multiple endocrine neoplasia 4	Autosomal dominant	*CDNKI B*
Adrenocortical carcinoma [68]	Beckwith-Wiedemann syndrome	Imprinting and growth abnormalities	Various genetic or epigenetic defects on chromosome 11p15.5
	Li-Fraumeni syndrome	Autosomal dominant	*TP53*
	Multiple endocrine neoplasia type 1	Autosomal dominant	*MEN1*
	Neurofibromatosis type 1	Autosomal dominant	*NF1*
Pheochromocytomas and paragangliomas [71]	SDH deficiency syndrome	Autosomal dominant (SDHD with paternal inheritance)	*MAX, SDHx* genes,* TMEM127, FH*
	Multiple endocrine neoplasia 2 A (Sipple syndrome)/B (multiple mucosal neuroma syndrome)	Autosomal dominant	*RET* (codon 634 and 918 respectively)
	Neurofibromatosis type 1	Autosomal dominant	*NF1*
	Von Hipple Lindau	Autosomal dominant	*VHL*
Thoracic tumors			
Pleuropulmonary blastoma [9]	DICER-1 syndrome	Autosomal dominant	*DICER1*
Carcinoid tumors [57]	Multiple endocrine neoplasia 1	Autosomal dominant	*MEN1*
	Von Hipple Lindau	Autosomal dominant	*VHL*
Skin tumors			
Nevoid basal cell carcinomas [37]	Nevoid basal cell carcinoma syndrome (Gorlin-Goltz syndrome)	Autosomal dominant	*PTCH1, SUFU*
Sebaceous neoplasms [80]	Muir-Torre subtype of Lynch syndrome	Autosomal recessive	*MLH1, MSH2, MSH6, PMS2, MYH*
Skin cancers with UV signature [78]	Xeroderma Pigmentosum	Autosomal recessive	*XP* genes (*A, B, D, E, F, G, V*)
Melanomas [78, 79]	BAP1 tumor predisposition syndrome	Autosomal dominant	*BAP1*
	Xeroderma Pigmentosum	Autosomal recessive	*XP* genes (*A, B, D, E, F, G, V*)

This table gives an overview of cancer predisposition syndromes encompassing specific tumor types, including their predominant mode of inheritance and the most relevant genes or chromosomal alterations that are involved in these syndromes. A complete representation of the genetic background and detailed discussion of inheritance mode is beyond the scope of this pathology-oriented review, and the reader is referred to specialized literature for this purpose

## The role of the pediatric pathologist in pediatric cancer predisposition syndromes

Histopathology, molecular analysis, clinical genetic analysis, and counselling should ideally be performed in highly specialized tertiary referral centers, where findings are integrated and discussed in multidisciplinary tumor boards. Detection of CPS should start in the clinic, where certain findings (such as mucosal neuromas in multiple endocrine neoplasia type 2B) may alert the clinician to a certain CPS. Table 2 provides a list of these non-tumoral lesions and cancer types [3], which is comprehensive but subject to change as developments in this field are rapid. Referral of patient and family to a clinical geneticist and additional genetic analysis should follow.

**Table 2:** Non-central nervous system solid tumors and non-tumoral lesions (when applicable) warranting referral to the clinical geneticist

Bone and soft tissue
Cardiac rhabdomyoma
Cardiac myxoma
Rhabdoymyosarcoma
Anaplastic
Botryoid-type embryonal
Desmoid tumor
Malignant rhabdoid tumor
Nasal chondromesenchymal hamartoma
Osteosarcoma
Psammomatous melanotic schwannomas
* Non-tumoral*
Mucosal neuroma
Genital system
Gynandroblastoma
Juvenile granulosa cell tumor
Large cell calcifying Sertoli-Leydig cell tumor
Ovarian Sertoli-Leydig cell tumor
* Non-tumoral*
Gonadoblastoma
Uterine leiomyoma
Urinary system
Cystic nephroma
Nephroblastoma (Wilms tumor, when bilateral/multifocal)
Renal angiomyolipoma
Renal cell carcinoma
Anaplastic sarcoma of the kidney
Urothelial cell carcinoma
* Non-tumoral*
Renal medullary dysplasia
Nephrogenic rests
Digestive tract
Gastrointestinal cancer
Gastrointestinal stroma tumor (GIST)
Hepatoblastoma
Endocrine system
Adrenocortical carcinoma
Medullary thyroid cancer
Parathyroid carcinoma
Pheochromocytoma/paraganglioma Thyroid tumor of uncertain histogenesis (formerly classified as cribriform-morular variant of papillary thyroid cancer)
* Non-tumoral*
C-cell hyperplasia
Thyroid follicular nodular disease Adrenal cytomegaly
Adrenal medullary hyperplasia
Primary pigmented nodular adrenocortical disease
Thoracic
Carcinoid tumor
Pleuropulmonary blastoma
Skin
Melanoma
Miscellaneous
Endolymphatic sac tumors
Hemangioblastoma
Retinoblastoma
* Non-tumoral lesions*
Odontogenic keratocysts (multiple)

Pathologists should also be aware of the association of certain tumor types and CPS to alert clinicians when necessary. Ideally, every pediatric malignancy should be subjected to molecular analysis, either by targeted next-generation sequencing (NGS) on the basis of a panel of known cancer-related genes or by whole exome sequencing. In addition, fusion gene analysis should be done by RNA sequencing or multiplex fusion assays. Close collaboration between molecular biologists and pathologists will ensure quality control and representativeness of the sample, interpretation of molecular findings, and the generation of an integrated pathology report. Again, specific molecular findings may warrant clinical genetics referral, especially when findings suggest the presence of a germline abnormality.

## CPS-associated tumors and their histology

### Peripheral neuroblastic tumors

Peripheral neuroblastic tumors (pNTs) are derived from neural crest cells, which in their most primitive phase are totipotential and closely resemble stem cells. pNTs encompass a spectrum of neuroblastic differentiation from the undifferentiated neural crest morphology, to the fully differentiated ganglion cell. Histologically pNTs are classified according to the Shimada classification/International Neuroblastoma Pathology Classification System, based on the presence of neuropil and the percentage of differentiating neuroblasts. About 80% of patients with pNTs present with the most undifferentiated form of pNTs: neuroblastoma. This is also the most common solid extracranial tumor in the first year of life.

Most neuroblastomas occur sporadically. Familial pTNs are extremely rare. Only 1–2% show hereditary predisposition, with *ALK* and *PHOX2B* being the most common genes with underlying and highly penetrant variants in patients with familial pNTs. An even smaller subset of pNTs arises as part of a CPS (e.g., Noonan syndrome, Neurofibromatosis type 1 (NF1), Beckwith-Wiedemann syndrome (BWS), and Li-Fraumeni syndrome, Table 1). Many sporadic neuroblastomas are driven by amplification of *MYCN* and segmental chromosomal alterations (including 1p and 11q loss of heterozygosity, and 17q gain). In about 10% of sporadic tumors, *ALK* amplification is seen, which is often co-amplified with *MYCN*. Determining the underlying drivers of neuroblastoma tumorigenesis provides important information about prognosis (*MYCN* amplification is associated with aggressive clinical behavior) and precision medicine (*ALK* mutations). Extensive reviews of the molecular alterations seen in neuroblastomas can be found in the literature [4].

### Bone and soft tissue tumors

Pediatric musculoskeletal tumors comprise a wide histological spectrum and represent a diagnostic challenge for the pediatric pathologist in daily practice. While these tumors often occur sporadically, benign soft tissue tumors (such as leiomyomas in the context of hereditary leiomyomatosis and renal cell cancer (HLRCC) [5]) and sarcomas (e.g. osteosarcoma in Li-Fraumeni syndrome [6]) can be part of a CPS (Table 1). Of note, CPS are less frequently seen in sarcomas that are caused by specific gene fusions (e.g., Ewing sarcoma, alveolar rhabdomyosarcoma) [6]. As osteosarcoma and rhabdomyosarcoma are the most common malignant pediatric sarcomas, these will be discussed below.

#### Osteosarcoma

**Fig. 2:**
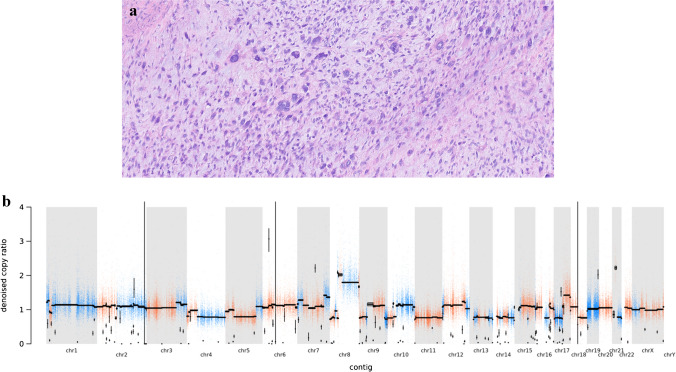
Nine-year-old boy with osteosarcoma in the context of Li-Fraumeni syndrome with germline *TP53* pathogenic variant. **A** Tumor histology showing extensive anaplasia of tumor cell nuclei in myxoid background. **B** Copy number variation profile typical of TP53-related osteosarcoma with numerous chromosomal gains and losses

Osteosarcoma is the most common bone tumor in children and adolescents, and is associated with a CPS (often seen in patients with Li-Fraumeni, but also in hereditary retinoblastoma syndromes, Table 1) in approximately 10% of affected children [6]. Young age at presentation, bilateral/multifocal disease, and metachronous tumors should point the clinician towards the possibility of a CPS. Histologically, these osteosarcomas do not differ from sporadic variants and are characterized by the presence of neoplastic osteoid/bone and malignant osteoblasts, generally with anaplastic features (Fig. 2). Osteosarcomas can be categorized into several subtypes based on their anatomical localization (central or peripheral) and histology (e.g., conventional, telangiectatic, small-cell).

#### Rhabdomyosarcomas (RMS)/DICER1-related sarcomas

Rhabdomyosarcomas are the most frequent pediatric soft tissue tumors. Histologically, they comprise the following subtypes: embryonal rhabdomyosarcoma (eRMS), alveolar rhabdomyosarcoma (aRMS), spindle cell/sclerosing rhabdomyosarcoma (scsRMS), and pleomorphic rhabdomyosarcoma. Of these subtypes, eRMS is the most common histological subtype in the context of a CPS. Recent studies point towards a new distinct molecular subtype: *DICER1*-related sarcomas primarily located in the (female) genitourinary tract, that have some similarities to eRMS. This entity will be discussed separately.

eRMSs are associated with a wide variety of CPS (Table 1) [7]. This highlights the importance of genetic testing in addition to standard NGS panels (which do not usually include germline variants) in patients without any other tumors or phenotypical characteristics pointing towards a CPS. Pediatric eRMS is a variably cellular tumor, comprised of primitive mesenchymal cells with spindle to round cell morphology, showing various stages of myogenesis with the presence of scattered differentiated rhabdomyoblasts. Two subtypes include botryoid eRMS and anaplastic eRMS (large atypical and multipolar mitotic figures, marked nuclear enlargement, and nuclear hyperchromasia), in which anaplasia may be focal or diffuse. Anaplastic eRMS has an association with germline *TP53* variants, hence Li-Fraumeni syndrome [8].

The association between germline *DICER1* pathogenic variants and pleuropulmonary blastoma (PPB) in the lung is well known. This malignant tumor was previously thought to be a rhabdomyosarcoma due to the presence of histological rhabdomyoblastic features. Later, PPB was characterized as a distinct entity in the context of *DICER1-*syndrome (vide infra [9]). Patients with *DICER1*-syndrome also show tumors (both benign and malignant, Fig. 1) at extra-pulmonary sites. Histologically, *DICER1-*related sarcomas show a morphological spectrum ranging from primitive cells (e.g., small blue round cells, spindle cells) to large pleomorphic (anaplastic) cells, arranged in a cambium layer underneath epithelial surfaces [10], and frequently display the characteristic presence of heterologous differentiation in the form of multiple foci of cartilage and sometimes osteoid [11, 12]. In addition, they may also show various degrees of rhabdomyoblastic differentiation, showing some similarities to eRMS [11]. However, *DICER1*-related sarcomas, in the genitourinary tract (mostly uterine corpus and cervix [13]) and elsewhere (including PPB), have been shown to carry strong morphological and molecular overlap regardless of their site of origin.

#### Desmoid-type fibromatosis

Pediatric desmoid tumors (DTs) are relatively rare neoplasms, predominantly located in the extremities. They may be the first clue towards a CPS (familial adenomatous polyposis (FAP)/Gardner syndrome). The majority of pediatric DTs result from germline variants in APC, which lead to oncogenic activation of the Wnt/β-catenin signaling pathway via accumulation of nuclear β-catenin [14]. The immunohistochemical accumulation of nuclear β -catenin (80% of cases) is helpful for diagnosis, as it may help to differentiate DTs from other fibroblastic proliferations. Gardner fibromas are important to mention, as they also occur in the context of familial adenomatous polyposis (FAP)/Gardner syndrome and can transform into DTs [15].

#### Gastrointestinal stromal tumors (GIST)

Pediatric GIST are associated with SDH deficiency syndrome, neurofibromatosis type 1, and familial GIST syndromes characterized by *KIT* or *PDGFRA* variants. In contrast to adult (*KIT* related) GIST that show spindle cell morphology, pediatric GIST often have epithelioid and mixed growth patterns, in addition to multifocality and a multinodular and plexiform growth pattern, which is related to the presence of SDH deficiency or *PDGFRA* variants [16, 17]. In GIST with *KIT* pathogenic variants, diffuse hyperplasia of interstitial cells of Cajal and skin pigmentation disorders are seen [18]. This is in contrast to GIST with *PDGFRA* pathogenic variants, which do not show diffuse hyperplasia but are associated with gastrointestinal inflammatory fibroid polyps, fibrous tumors, and disorders of mast cells [18].

#### Peripheral nervous system tumors

*Pediatric schwannomas* are most frequently seen in patients with neurofibromatosis type 2 (NF2) and to a lesser extent in patients with schwannomatosis (*SMARC-B1* and *LZTR1*; Table 1) [19]. They are benign tumors originating from the myelin-producing Schwann cells covering the peripheral nerve (usually cranial nerve 8 in patients with NF2 when occurring intracranially). Histological features more commonly seen in CPS-associated schwannomas include whorl formation and involvement of multiple nerves in NF2 and “peritumoral edema, myxoid changes, intraneural growth, and intratumoral nerve fibers” in schwannomatosis [20]. Schwannomas are also part of the Carney complex and are then commonly located in the upper gastrointestinal tract (esophagus and stomach), and paraspinal sympathetic chain, and have a characteristic histology (psammomatous melanotic schwannomas).

Neurofibromas are frequently seen in neurofibromatosis (most often type 1 (NF1) but also sporadically in NF2) and often involve peripheral nerves [19]. Subtypes include cutaneous, plexiform, intraneural, and localized forms of which the first two are strongly associated with NF1. Histologically, they are composed of multiple cell types (including a neural and non-neural/fibrous component); while the cutaneous forms are benign, plexiform neurofibromas do have malignant potential and can progress towards a malignant peripheral nerve sheath tumor (MPNST).

*Malignant peripheral nerve sheath tumors (MPNST)* in childhood are seen in individuals with NF1, but also in other CPS such as Li-Fraumeni syndrome (Table 1) [19]. A subset of these tumors represents malignant transformation from a plexiform neurofibroma. Histologically, MPNSTs in the context of a CPS are mostly high grade, with a conventional spindle cell morphology [21].

### Genital system

#### Gonadoblastoma

Gonadoblastomas are rare tumors, representing an in situ form of malignant germ cell tumors (GCTs). They are most often seen in patients with gonadal dysgenesis (as seen in Frasier, Swyer, or Turner syndrome). Knowledge about macroscopic and microscopic normal and abnormal gonadal development is necessary to adequately diagnose these tumors. This is beyond the scope of this review, but was described in detail by others [22–24].

#### Extragonadal germ cell tumors (eGCTs)

eGCTs are frequently occurring tumors in the pediatric population, especially in adolescents and young adults. While primarily localized in the gonads, they can also occur at extragonadal sites [25]. The presence of extragonadal GCTs in young patients should raise suspicion of an underlying CPS with mediastinal germ cell tumors being described in patients with Klinefelter syndrome [26] and sacrococcygeal teratomas/germ cell tumors in patients with Schinzel-Giedion syndrome [27, 28]. Other important CPS associated with eGCT include Swyer and Currarino syndrome (Table 1) [25, 29, 30].

#### Non-germ cell tumors

*Small-cell carcinoma of the ovary hypercalcemic type (SCCOHT)* is a very rare, highly aggressive tumor and presents at a young age (usually < 15 years) [31]. SCCOHT bears great resemblance to malignant rhabdoid tumors at other sites, and is seen in patients with rhabdoid tumor predisposition syndrome (RTPS; generally type 2 with germline mutations in *SMARCA4*). Histology shows a relatively monotonous undifferentiated tumor with immunohistochemical loss of nuclear BRG1 staining. However, BRG1 staining might be retained in rare cases that are *SMARCA4*-wildtype, and instead show mutations in *SMARCB1* (INI1, RTPS type 1) with loss of immunohistochemical INI1 staining [32].

#### Ovarian sex cord-stromal tumors

These types of tumors are uncommon in children and account for approximately 10–15% of pediatric ovarian tumors [33].

*Ovarian Sertoli-Leydig cell tumors (OSLCT)* comprise roughly 1–2% of pediatric ovarian tumors, but are more frequently seen in association with *DICER1* variants [34]. Histologically, OSLCTs in *DICER1* patients show moderate to poor differentiation, and are characterized by the presence of heterologous elements (analogous to other *DICER1-*related tumors) and/or retiform growth patterns [35]. Typically, Leydig cells are sparse, and when present, they are located at the tumor periphery [36].

*Ovarian fibromas* in the pediatric population are rare. When occurring, the possibility of Gorlin-Goltz syndrome should be considered. Ovarian fibromas in the context of this CPS are often bilateral, calcified, and nodular [37].

### Urinary system

Renal tumors are relatively frequent in childhood and adolescence. During childhood, nephroblastoma (also known as Wilms tumor) is the most common renal malignancy, accounting for approximately 90% of all pediatric renal tumors. Other renal malignancies include renal cell carcinomas (mostly seen in adolescents) and less commonly seen tumors such as malignant rhabdoid tumor of the kidney (MRTK), clear cell sarcoma, and congenital mesoblastic nephroma.

#### Nephroblastoma (Wilms tumor)

**Fig. 3:**
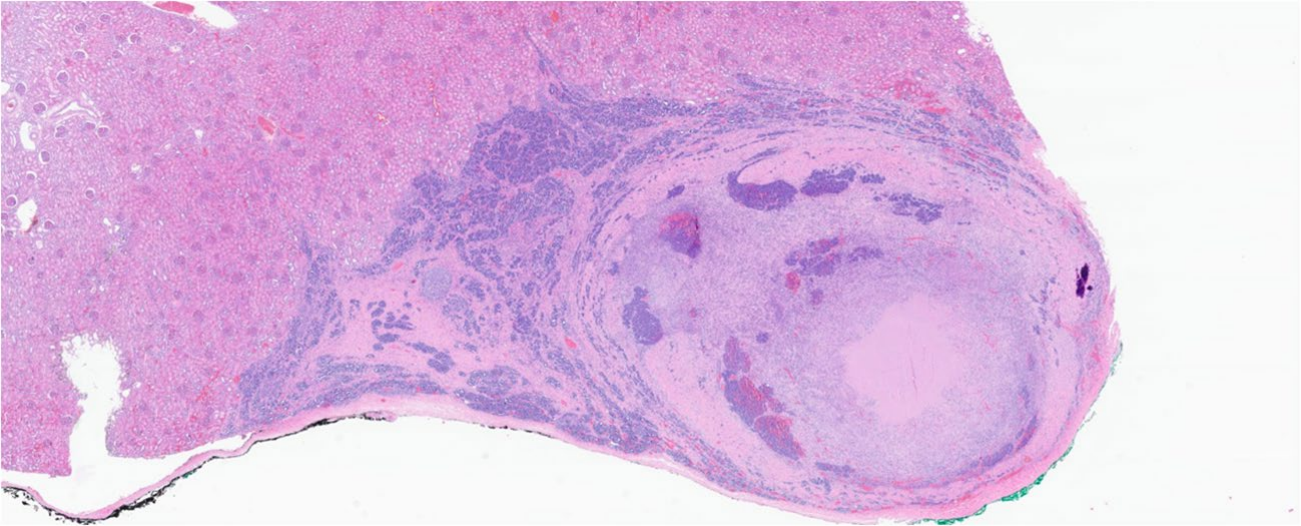
Hematoxylin and eosin-stained section of diffuse hyperplastic perilobar nephrogenic rests in the context of nephroblastomatosis in a patient with Beckwith-Wiedemann syndrome

About 30% of Wilms tumors are associated with a CPS. Although *WT1-*mutation-associated syndromes are the most common, Wilms tumors can be seen in a wide variety of CPS (Table 1) [38]. Treatment of nephroblastoma varies between Europe and North America. While the SIOP studies from Europe (including UMBRELLA) advocate preoperative chemotherapy, the North American NWTSG/COG guideline advises upfront surgery. These different regimes result in different histological and staging criteria. However, in both systems, identification of anaplasia is of great importance, as diffuse anaplasia significantly influences prognosis and treatment. Anaplasia is defined histologically as the presence of aneuploid hyperchromatic nucleomegaly, with atypical and/or multipolar mitoses and marked nuclear enlargement of at least three times the size of adjacent tumor nuclei. Tumors with diffuse anaplasia often show TP53 mutations [39], which are secondary events corresponding to the low incidence of nephroblastomas in patients with Li-Fraumeni syndrome. Histology might be helpful in identifying familial Wilms tumors, with histologic clues including (1) the presence of nephroblastomatosis (Fig. 3) and (2) predominant histological subtype (stromal-predominant variants are associated with *WT1* mutation; epithelial predominant with *TRIM28* mutations). For the latter, specific immunohistochemistry with the TRIM28/KAP1 antibody showing loss of expression may indicate the presence of a *TRIM28* mutation. In addition, the presence of medullary ray nodules in the pre-existent renal medulla may be a clue for the presence of Beckwith-Wiedemann syndrome [40, 41]. For an extensive review on guidelines and histology, the reader is referred to the literature [42].

#### Renal cell carcinoma (RCC)

RCCs in children are often associated with CPS (von Hippel Lindau (VHL), hereditary papillary renal cell carcinoma (HPRC), hereditary leiomyomatosis and renal cell cancer (HLRCC), PTEN-hamartoma tumor syndrome, SDH deficiency syndromes). Age at onset serves as an important clue for genetic testing, just as bilateral/multifocal papillary tumors or other associated extrarenal tumors (intra- or extracranial hemangioblastoma (VHL) and leiomyomas (HLRCC) [43]). The pathologist has an important role in recognizing the possibility of a renal cell carcinoma in the context of a CPS, as the histology can provide various clues, such as a clear cell morphology that is mostly seen in patients with VHL. Immunohistochemistry may also direct the diagnosis, such as loss of FH-staining in HLRCC-associated RCC and loss of SDHA or SDHB staining in SDH-deficient RCC in the context of SDH deficiency syndrome. For more detailed information on the morphological traits of these subtypes, the reader is referred to other sources [44].

#### Malignant rhabdoid tumor of the kidney (MRTK)

MRTK is a highly aggressive tumor seen in the context of RTPS, generally in RTPS type 1 (*SMARCB1/INI1* mutations) and rarely in RTPS type 2 (*SMARCA4* mutations) [45]. Although classically characterized by infiltrating sheets of discohesive cells with eccentric nuclei and abundant eosinophilic cytoplasm (“rhabdoid appearance”) with brisk mitotic activity and necrosis, these tumors show extensive histological variability, including epithelioid, small cell, and myxoid growth patterns. Therefore, pathologists should have a low threshold for ancillary tests in addition to morphology to ensure adequate diagnosis of MRTK. In addition to the kidney, rhabdoid tumors occur at various locations throughout the body. They may also be seen in the central nervous system, in which they are called atypical teratoid rhabdoid tumors (ATRT), sharing histological and molecular features with MRTKs.

### Digestive tract (alimentary tract, liver, and pancreas)

#### Liver tumors

Compared to other solid embryonal pediatric tumors (such as neuroblastoma and nephroblastoma), liver tumors in children are relatively rare. The most common liver tumors are hepatoblastomas (HBs), which present at a young age (median age at diagnosis: 3 years), and hepatocellular carcinomas (HCCs) that primarily occur in adolescents. While HBs have a clear link with CPS (approximately 1/3rd arise in the context of a CPS), this association is less pronounced for pediatric HCCs. Histological differentiation between HBs and HCCs may be challenging due to morphological overlap but is essential due to differences in treatment (chemotherapy versus upfront surgery) [46].

**Fig. 4:**
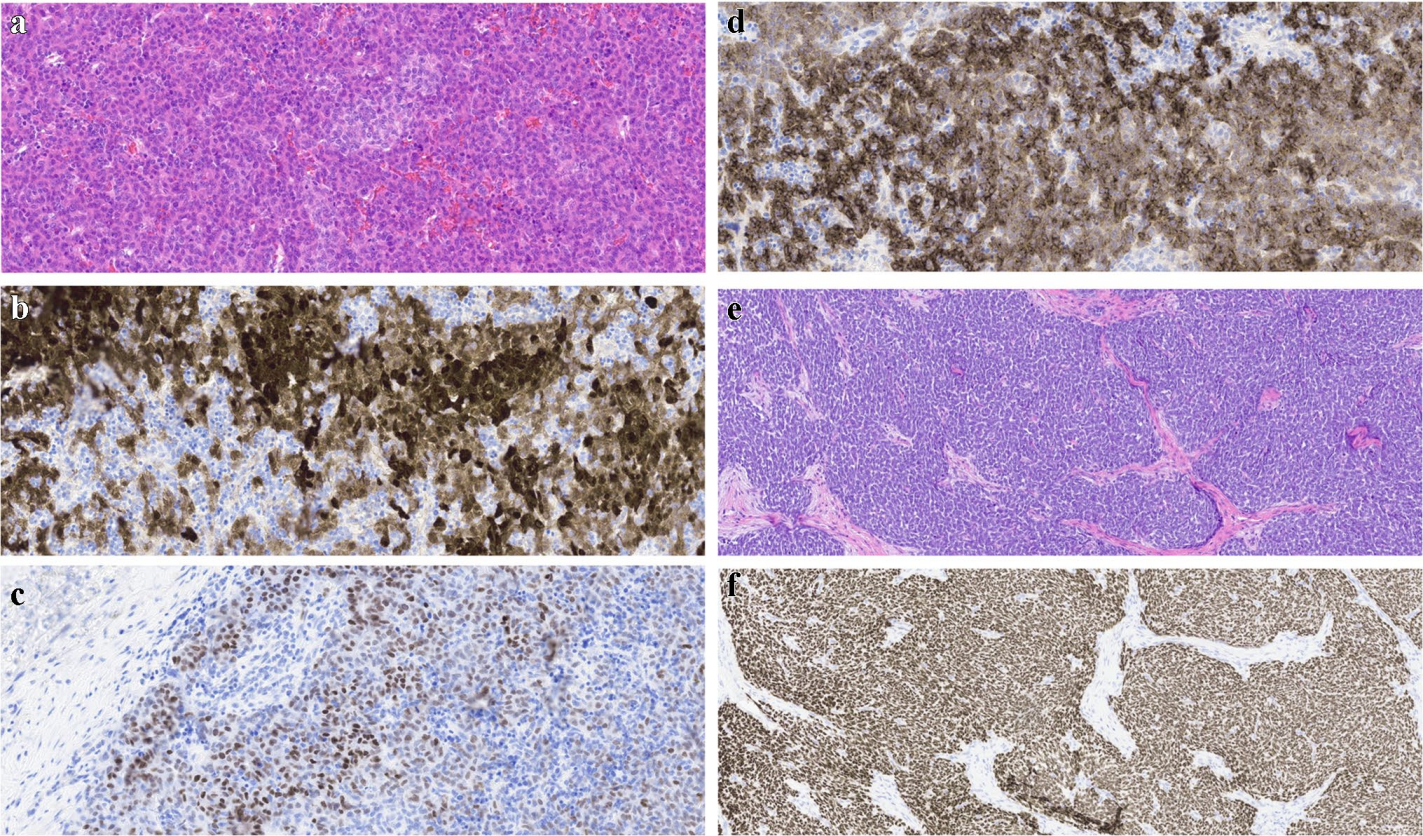
Four-year-old boy with Beckwith-Wiedemann syndrome, presenting with liver tumor at age 2 months, diagnosed as fetal epithelial hepatoblastoma. A detail of combined fetal and embryonal differentiation can be seen (**A**), with the corresponding area positive for cytoplasmic and nuclear staining with beta-catenin (**B**), positive for nuclear staining mostly in embryonal cells (**C**), and cytoplasmic staining in all tumor cells for glypican 3 (**D**). At 2 years and 9 months follow-up, he presented with a right renal mass, suspected of hepatoblastoma relapse. However, the tumor was found to be a non-anaplastic Wilms tumor, as can be seen on the HE staining (**E**), supported by nuclear positivity for WT1 (**F**)

*Hepatoblastomas (HB)* can be seen in CPS such as BWS and FAP [47, 48] (Fig. 4). Histologically, hepatoblastomas reproduce the developmental stages of the liver and consist of epithelial (fetal, embryonal, pleomorphic, small-cell undifferentiated, cholangioblastic, macrotrabecular patterns) and/or mesenchymal components (mostly osteoid). When more than one heterologous element is present (including endoderm and neuroectodermal derivatives), they are classified as teratoid hepatoblastomas. Adequate diagnosis is of great importance as the various subtypes are associated with different prognoses and require different treatments. Central in the pathogenesis of HB are mutations in the APC/beta-catenin pathway. Molecular analyses might also provide a clue to distinguishing sporadic tumors from familial cases, with beta-catenin mutations (especially mutations in exon 3 [49]) mainly being present in sporadic cases, while APC mutations might point to a familial cause (HBs in the context of FAP [47]).

*Hepatocellular carcinoma (HCC)* comprises approximately 20% of all pediatric malignant liver tumors and is often present in older children/adolescents, carrying a poor prognosis. Two subcategories can be identified: those occurring in livers with and without underlying (chronic) diseases. The former mainly include metabolic/genetic conditions (e.g., hereditary tyrosinemia, progressive familial cholestasis), but also developmental disorders (such as Alagille syndrome and Caroli disease [50]) that result in the accumulation of toxic metabolites that predispose to the formation of HCCs. CPS are hardly ever seen as an underlying cause for HCC, with few case reports on HCC in patients with overgrowth syndromes (e.g., Sotos [51] and Simpson-Golabi-Behmel syndrome [52]). A distinct form of HCC worth mentioning is the fibrolamellar variant of hepatocellular carcinoma (FLHCC). Patients with these tumors present at a young age without underlying chronic liver disease. Immunohistochemically, these tumors have a characteristic signature, showing co-expression of CK7 and CD68 (due to the abundance of lysosomes in tumor cells [53]). Molecular tests show a *DNAJB1::PRKACA* fusion in the vast majority of cases, except for FLHCC arising in the context of Carney complex which is characterized by *PRKAR1A* mutations [54].

#### Pancreatic tumors

Pancreatic tumors in children are rare. *Pancreatoblastoma (PB)* is the most common malignant pediatric pancreatic tumor, and is associated with CPS such as BWS and FAP. PB shows genetic resemblance to HB, also having molecular alterations in the APC/β-catenin pathway [55]. *Cystic lesions* can also be seen in childhood, and can occur in the context of VHL [56]. There are no specific histologic clues to distinguishing pancreatic neoplasms in the context of a CPS. Neuroendocrine neoplasms of the pancreas will be discussed in the section below.

#### Neuroendocrine neoplasms (NENs)

NENs in children are very rare and can be seen as part of a CPS (e.g., VHL, multiple endocrine neoplasia type (MEN) 1 and 2, tuberous sclerosis complex (TSC), and NF1) [57]. This section focuses on NENs in the gastrointestinal tract; other NENs (such as pulmonary carcinoid tumors) will be addressed separately. NENs can be categorized into neuroendocrine tumors (NETs) and poorly differentiated neuroendocrine carcinomas (NECs). NETs are subsequently graded based on their mitotic count and Ki-67 index into grades 1, 2, and 3 (less than 2 mitosis per 2mm2 or Ki67 less than 3%; 2–20 mitosis per 2mm2 or Ki67 between 3 and 20%; and > 20 mitosis per 2mm2 or Ki67 greater than 20%). A number of features are direct towards a CPS-associated NEN. First, pediatric NETs in the context of a CPS are mostly located in the pancreas and duodenum [57], and can be functioning (often gastrinomas) or non-functioning. Second, NENs in the context of for example VHL are histologically characterized by stromal collagen bands and clear cell morphology [58].

### Endocrine system

Neoplasms of the endocrine glands are associated with a wide spectrum of CPS, including MEN (types 1, 2, 4), Li-Fraumeni syndrome, BWS, and SDH deficiency syndrome (Table 1). In this section, the most commonly associated neoplasms will be discussed.

#### Thyroid tumors

Thyroid cancer is seen in a variety of CPS, of which FAP, PTEN-hamartoma tumor syndrome, Li-Fraumeni, *DICER1* syndrome*,* Carney complex, and MEN2 are among the most important ones. Thyroid tumors occurring in the context of a CPS can be derived from both follicular cells (follicular neoplasms and papillary neoplasms) as well as calcitonin-producing cells (medullary thyroid carcinoma). While follicular thyroid carcinomas in children often occur unilaterally and are minimally invasive (not widely invasive), papillary thyroid carcinomas (PTC) have an increased risk of bilateral presentation and a more aggressive course [59]. Medullary thyroid carcinomas in children and adolescents are almost exclusively seen in patients with MEN2.

**Fig. 5:**
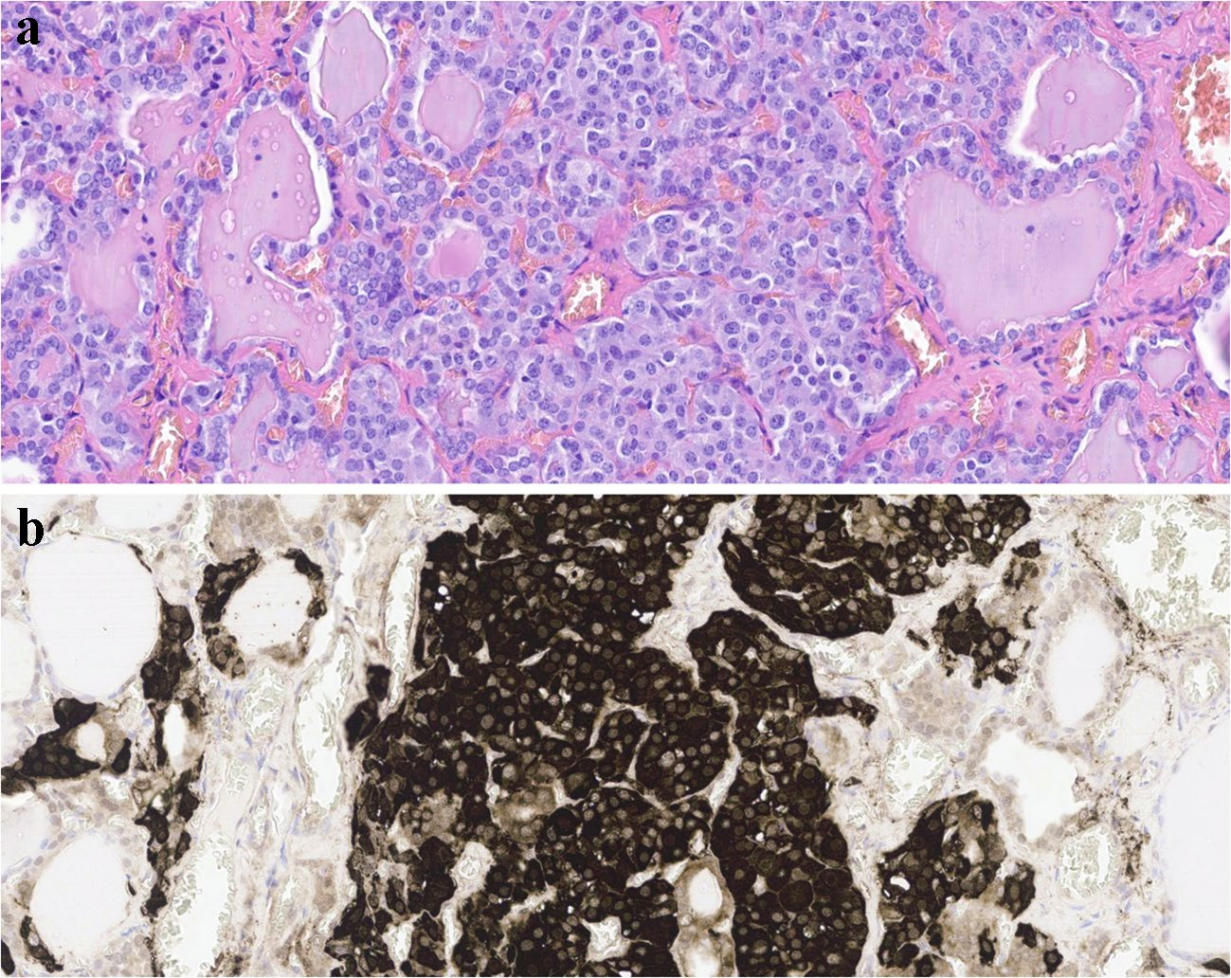
**A** Hematoxylin and eosin-stained section of prophylactic thyroidectomy showing proliferation of epithelial cells, suggestive of C-cell hyperplasia; **B** corresponding calcitonin immunostaining showing positive staining of the proliferating epithelial cells, confirming the diagnosis of C-cell hyperplasia in a patient with MEN2A syndrome. This proliferation falls short of incipient medullary thyroid carcinoma, for lack of infiltrative growth, lack of desmoplastic stroma, and lack of amyloid deposition

Histologically, there are several clues that point towards CPS-related thyroid pathology. In addition to follicular carcinomas, patients with PTEN-hamartoma tumor syndrome characteristically show multiple adenomatous nodules (including adenolipomas and microadenomas) in a background of lymphocytic thyroiditis and/or C-cell hyperplasia [60–62]. Thyroid pathology is also often seen in *DICER1* patients, with early onset (< 10 years) thyroid follicular nodular disease with the presence of clonal nodules (in contrast to polyclonal nodules in patients without *DICER1*) as the most prominent phenotypic feature [63]. *DICER1*-related malignant thyroid tumors are often indolent, but also include poorly differentiated carcinoma and thyroblastoma. In patients with MEN2, the thyroid shows C-cell hyperplasia, for which prophylactic thyroidectomy is recommended [64] since this evolves into medullary thyroid carcinoma, the most frequent thyroid tumor in this syndrome (Fig. 5).

Cribriform-morular thyroid carcinoma is also worth mentioning for its characteristic histological appearance. In the WHO Blue Books, this tumor is classified as a “thyroid tumor of uncertain histogenesis.” For additional distinct thyroid pathology pointing towards a CPS, the reader is referred to other literature [65].

#### Parathyroid neoplasms (PNs)

The characteristic CPS associated with PNs is multiple endocrine neoplasia (types 1, 2, and 4). MEN1 often involves multiple glands; MEN2 tends to only affect one gland. Histologically, adenomas show fat-depleted, hypercellular parathyroid tissue often consisting of predominantly chief cells. Although pediatric parathyroid carcinomas (based on the histological presence of invasive growth and/or vascular invasion) almost never occur, they present in the setting of HRPT2 mutations leading to hyperparathyroidism-jaw tumor syndrome (HPT-JS). Therefore, a history of personal or familial parathyroid carcinoma requires evaluation for germline *HRPT2* mutation status which is important for early detection and removal of potentially malignant parathyroid neoplasms [66]. Adequate histology and ancillary testing may provide a clue, as certain morphological aspects (including sheet-like growth pattern, eosinophilia, nuclear enlargement, arborizing vasculature) and parafibromin deficiency on immunohistochemistry almost always point towards HPT-JS [67].

#### Adrenal tumors and tumors of the extra-adrenal paraganglia

*Adrenocortical carcinoma (ACC)* is only sporadically seen in children (0.2% of pediatric cancers). It is an aggressive tumor and one of the core cancer types of Li-Fraumeni syndrome, in which ACC already occurs at a young age [68]. Endemic areas for ACC exist, with a 10- to 15-fold increase in ACC in Southern Brazil, due to the high prevalence of a specific germline TP53 variant [R337H] [69]. Regardless of family history, diagnosing ACC in children should prompt genetic testing as de novo variants, or incomplete penetrance of TP53 germline mutations might explain their occurrence in the absence of a positive family history [70]. Other CPS in which ACC can be seen include BWS, MEN1, and NF1 (Table 1, Fig. 1).

*Pheochromocytomas (PCC)* and *paragangliomas (PGL)* have the strongest association with germline pathogenic variants [71]. They are often the presenting and sometimes only feature directing towards a hereditary cause (e.g., in MEN2A/B and VHL), and the presence of bilateral or multifocal tumors and adrenal medullary hyperplasia should alert the clinician towards PCC/PGL in the context of germline disease. PCC/PGL are derived from chromaffin cells (located in the adrenal medulla and extra-adrenal paraganglia, respectively) and are all considered malignant. In some cases, metastases (often seen in combination with *SDHB* germline variants) may occur [72]. While certain histological clues have been described in VHL-related tumors, such as the occurrence of clear cell morphology and high vascularity, the correlation between morphology and other CPS is less obvious [73].

### Thoracic tumors

Pediatric pulmonary neoplasms are rare, and often represent metastases from extra-pulmonary tumors, such as osteosarcomas, Wilms tumors, and rhabdomyosarcomas. Primary pulmonary neoplasms in childhood are even less frequently seen, with a reported incidence of 0.03%, and include carcinoid tumors and pleuropulmonary blastoma (PPB). Carcinoid tumors are mostly seen at an older age (age at diagnosis 15–19 years old), while PPB is an aggressive malignancy that predominantly affects infants and young children [74].

#### Pleuropulmonary blastoma

**Fig. 6:**
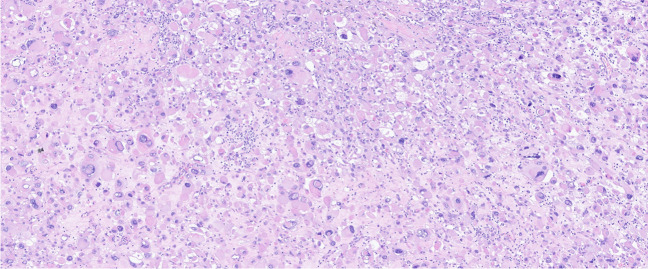
Typical histology of pleuropulmonary blastoma in a DICER1 patient, showing profound nuclear pleomorphism in tumor cells with ample eosinophilic cytoplasm suggesting rhabdomyoblastic differentiation

PPB is the archetypical malignancy associated with germline pathogenic *DICER1* variants [9]. PPB can be divided into subtypes based on macroscopic features, which vary from entirely cystic tumors to completely solid lesions. Type I PPB is completely cystic, and can be found antenatally. Diagnosis might be difficult, and misdiagnosis in the form of other congenital cystic lesions (such as the spectrum of lesions associated with bronchial obstruction deformation sequence, formerly referred to as “congenital pulmonary airway malformation”) may occur. Cysts represent expanded airspaces lined by alveolar or bronchiolar epithelium with dispersed subepithelial foci of primitive mesenchymal cells with or without rhabdomyoblastic differentiation (Fig. 6). Small nodules of cartilage may also be present. Type I is also purely cystic, but lacks the primitive mesenchymal elements, and is regarded as a regressed form of type I PPB. Type II and type III PPB also show solid parts, in which type III is completely solid while type II still shows cystic changes. The solid areas show one of the following elements: (1) nests consisting of primitive blastemal-like cells with nuclear atypia and abundant mitoses; (2) spindled, stellate, and ovoid cells with a background of myxoid stroma; (3) compact foci with a spindle cell or fibrosarcomatous growth pattern; (4) chondroid nodules (often immature, or of malignant appearance). The differential diagnosis with rhabdomyosarcoma might be difficult, especially on core biopsies [75].

#### Carcinoid tumors

Carcinoid tumors are the most common primary pulmonary malignancy in children, and are associated with a number of CPS, such as MEN1 and VHL [57]. Grading these neuroendocrine tumors by measuring cellular proliferation (mitotic count/Ki67) is important and reflects their biological behavior. Neuroendocrine tumors are described in more detail in the section on NENs in the digestive tract.

### Skin tumors

Many CPS present with cutaneous findings (e.g., mucocutanous hyperpigmentation in Peutz-Jeghers, multiple café-au-lait spots in NF1, and constitutional mismatch repair deficiency), and apparently, benign congenital skin lesions might be associated with malignant tumors such as neurovascular hamartomas and malignant rhabdoid tumors [76]. It is beyond the scope of this review to discuss all of these, and the reader is referred to the literature [77]. Primary pediatric skin tumors, however, are rare. Skin tumors associated with CPS include nevoid basal cell carcinomas as part of Gorlin-Goltz syndrome, sebaceous neoplasms in the Muir-Torre subtype of Lynch syndrome, and skin cancers with a UV signature in xeroderma pigmentosum. The latter syndrome may also be associated with melanomas [78]. Other CPS worth mentioning in the context of pediatric melanomas include hereditary melanoma and pancreatic cancer syndrome (or familial atypical mole-malignant melanoma syndrome) and BAP1 tumor predisposition syndrome [78, 79].

An in-depth discussion of the salient histopathological features of all these tumors is beyond the scope of this review. Pathologists should be aware of the association with certain CPS especially when multiple different tumors are present, which require additional immunohistochemical stains when required (such as MMR immunohistochemistry in children with sebaceous carcinoma, and BAP1 in the context of melanocytic lesions).

## Limitations of pathology to detecting a CPS

The diagnosis of any CPS is an interplay between clinicians, pathologists, and geneticists. None of these specialists will be able to call all CPS in a group of pediatric patients, even if these are pediatric cancer patients, unless systematic and extensive molecular analysis is done. However, in many settings, this is technically and financially impossible. Inherently, pathology has its limitations in recognizing CPS as well. Frequently, sporadic and CPS-related tumors do not show distinctive histopathological clues, or existing specific clues are not present in microscopic slides (such as medullary ray nodules in the kidney). In addition, not all pathologists are sufficiently familiar with the numerous CPS and the ever-growing number of tumor types that occur in each of them. Finally, ancillary techniques such as immunohistochemistry and molecular tests may provide important indications for the presence of a CPS (such as loss of SDHB expression in relation to SDH deficiency syndrome, or a high variant allele frequency for any given genetic variant). However, this is the case for a limited number of immunohistochemical tests and molecular analyses. Furthermore, the latter are primarily directed at the discovery of somatic gene abnormalities, and not germline abnormalities. Thus, referral to clinical genetics is imperative for certain tumor types or combinations of tumors, even in cases without supportive evidence for a CPS.

## Conclusion

The number and phenotypic diversity of cancer predisposition syndromes have grown significantly over the past decades. Likewise, their etiology and pathogenesis are increasingly being unraveled. This holds the promise that affected individuals may be diagnosed at an early age, leading to improved care, cure, and even prevention of tumor formation by tailored screening programs. While definitive diagnoses need to be provided by clinical geneticists on the basis of family history and germline genetic analysis, the first presentation of CPS is frequently through an initial tumor in an index patient. Pathologists have a crucial role in identifying tumor types or specific histological clues (whether precursor lesions or specific morphological patterns) to flag the probability of a CPS-related lesion, thereby allowing prompt referral of patients and families. This review provides a comprehensive overview of non-central nervous system solid pediatric tumors and their occurrence in CPS and delivers to interested pathologists the key information for their detection.
